# Compressive buttress compared with off-axial screw fixation for vertical femoral neck fractures in young adults: a prospective, randomized controlled trial

**DOI:** 10.1186/s13018-023-04493-y

**Published:** 2024-01-06

**Authors:** Bo-hao Yin, Chen-jun Liu, Matthew C. Sherrier, Hui Sun, Wei Zhang

**Affiliations:** 1 Shanghai Sixth People’s Hospital Affiliated to Shanghai Jiao Tong University School of Medicine, Shanghai, 200233 China; 2 Shanghai Sixth People’s Hospital, National Center for Orthopaedics, Shanghai, 200233 China; 3 Department of Orthopaedics and Physical Medicine, Medical University of South Carolina, Charleston, SC 29425 USA

**Keywords:** Femoral neck fracture, Screw fixation, Compression, Buttress, Complication

## Abstract

**Background:**

To compare the clinical outcomes of compressive buttress screw (CBS) fixation, a novel screw fixation strategy, to off-axial screw fixation (off-axial partial threaded cannulated screw, OPTCS) for vertical femoral neck fractures (FNFs) in young adults.

**Methods:**

A total of 146 adults younger than 55 years old with high-energy Pauwels type III FNFs were randomized to receive CBS fixation or OPTCS fixation. Primary outcomes were complication rates, including fixation failure, fracture nonunion, and avascular necrosis of the femoral head (ANFH) at 24 months after treatment. Fixation loosening, femoral neck shortening and varus collapse, patient function and quality of life using the Harris hip score (HHS), and EuroQol-5 dimensional-5 levels (EQ-5D-5L) questionnaire (including EQ-5D-5L and EQ-VAS) were assessed as secondary outcomes at 24 months.

**Results:**

CBS and OPTCS fixation groups were similar with regard to demographics at baseline. At 24 months, patients in the CBS fixation cohort had a significantly lower rate of fixation failure (10.5% vs. 25.0%, *p* = 0.041) and fracture nonunion (1.8% vs. 18.3%, *p* = 0.003) compared with patients who received OPTCS fixation. There was no difference in rate of ANFH (7.0% vs. 11.7%, *p* = 0.389) between groups. Additionally, patients managed with CBS fixation showed significantly less fixation loosening (19.3% vs. 58.3%, *p* < 0.001), less severe femoral neck shortening and varus collapse (10.5% vs. 25.0%, *p* = 0.007), higher HHS (93 vs. 83, *p* = 0.001) and more excellent grade (68.4% vs. 36.7%, *p* = 0.008), higher EQ-5D-5L (0.814 vs, 0.581, *p* < 0.001) and EQ-VAS (85 vs. 80, *p* = 0.002).

**Conclusion:**

CBS screw fixation confers significantly lower complication rate in addition to higher functional and quality of life outcomes for young adults with high-energy FNF compared with OPTCS fixation.

**Trial registration:**

This prospective, randomized controlled trial was approved by the institutional review board of our center, Ethics Committee of Shanghai sixth people's Hospital, and registered at www.chictr.org.cn (Approval Number: ChiCTR1900026283; Registered 29 September 2019—Retrospectively registered, https://www.chictr.org.cn/showproj.html?proj=43164).

## Introduction

Hip fractures are a commonly encountered traumatic injury in orthopedic practice that are associated with substantial morbidity, mortality, and costs [1–5]. Femoral neck fracture (FNF) is a typical representative of hip fracture, and its treatment often varies according to the age of the patient [6]. Although occurring at a lower incidence than in the elderly, FNF in the young population presents a unique clinical challenge for the orthopedic traumatologist due to higher postoperative functional demands for work or recreational activities. Additionally, FNF in young adult patients is typically associated with high-energy trauma and displaced fracture patterns, resulting in a biomechanically disadvantageous environment for fracture healing [7–10]. The high proportion of fixation failures resulting in reoperation has generated controversy about the most appropriate surgical treatment for FNF [7]. Regardless of the fixation method used, pooled incidences of complications after FNF fixation in young patients are common, including reoperation (18.0%), avascular necrosis of the femoral head (ANFH) (14.3%), and nonunion (9.3%) [7]. As such, treatment of FNF in young patients presents a clinical challenge when choosing the optimum fixation approach and is associated with significant complications [8,10–12].

The available internal fixation techniques for FNF are classified based on mechanism and type of fixation: traditional sliding compression fixation, angular stabilization fixation, length-stable implant fixation, medial buttress plate fixation, and off-axial screw fixation, etc. [13]. Except the traditional sliding compression fixation, including multiple parallel partial-threaded cancellous cannulated lag screw (PTCS) and dynamic/sliding hip screw (DHS&SHS) fixation [14], other fixations are not recommended as common treatments for FNF [8]. Although biomechanical and clinical studies have evaluated various options, the optimal fixation construct to allow for healing and prevention of complications after FNF is still unknown [10]. The PTCS fixation is a widely accepted technique with reported advantages of less tissue invasiveness and blood loss in addition to shorter hospital stay and operation time [15,16]. However, recent studies have shown high complication rates in vertical FNFs fixed with PTCS, attributed to poor biomechanical performance [12]. Therefore, off-axis screw configuration in which a horizontal or transverse screw implanted orthogonal to the vertical fracture line besides the PTCS, has been proposed as a surgical option for the treatment of vertical FNFs. Although clinical data are limited [17–19], studies have demonstrated superior biomechanical stability with off-axis screw configurations when compared with traditional PTCS fixation [19–24]. In particular, the use of OPTCS (three parallel screws plus with an off-axis trochanteric transverse screw) for the treatment of vertical FNFs has demonstrated promising clinical results in recent studies [19,25,26]. At this time, there is no definite agreement on the effectiveness of this new screw configuration for FNF treatment.

The fully threaded headless cannulated screw (FTHCS) was originally introduced as length-stable fixation implant to prevent the femoral neck shortening for the FNF treatment with the characteristics of normal head, cylindrical profile. Meanwhile, another kind of fully threaded screw, FTHCS possessing the specific features of tapered profile and variable pitch, fully threaded headless cannulated screw have been introduced [27]. It was shown by our previous biomechanical experiments and clinical study that comparing with conventional PTCS fixation, the new fixation configuration using FTHCS could not only supply better biomechanical stability but also reduce the complication rate significantly for vertical FNF [28–31]. In response to a recent clinical study demonstrating a distinctive failure model of medial migration and superior cut-out of the proximal screw in three FTHCSs fixation for vertical FNF in young patients [31], a novel screw configuration named compression buttress screw (CBS) fixation has been proposed [13]. In CBS fixation, two distal FTHCSs are combined with one proximal partial threaded cannulated screw (PTCS) in a regular triangular configuration. To the best of our knowledge, no prospective study has compared the clinical outcomes of these two configurations (CBS vs. OPTCS) on young patients with vertical FNFs. Thus, the objective of this prospective randomized, controlled trial was to evaluate the efficacy of CBS fixation with the OPTCS fixation on complication rates, hip function and life quality in young patients with vertical FNF. The null hypothesis was that there is no difference in complication rates, pain, or functional outcomes between groups at 24 months.

## Materials and methods

This prospective, randomized controlled trial was approved by the institutional review board of our center and registered at www.chictr.org.cn (ChiCTR1900026283). From January 2016 to July 2017, all adult patients younger than 55 years presenting to our level-I trauma center with FNFs were considered eligible for enrollment.

### Participants

Patients age younger than 55 years who suffered from FNFs with > 50° of verticality (Pauwels type III) [32] needing treatment with internal fixation were considered for inclusion. Exclusion criteria included patients with skeletal immaturity (age ≤ 16 years), pathological fractures, nonfresh fracture, re-fracture, polytrauma with an injury severity score (ISS) of > 16 [33], deformity or dysplasia affecting the lower extremities, rheumatologic or other immunopathologic arthritic disease of the hip, ANFH, uncontrolled diabetes mellitus, alcohol abuse, concurrent participation in another clinical study, fair or poor preoperative grade of function according to the Harris hip score (HHS) [34], and an inability to cooperate with treatment and follow-up. In patients who sustained multiple traumatic injuries with ISS ≤ 16, injuries were triaged for treatment based on clinical judgment; however, all FNF included in the study were treated within forty-eight hours after presentation. All participants provided written informed consent.

### Fracture management and treatment allocation

Upon presentation to the emergency department, demographic data including the mechanism of injury, medical history, and preinjury comorbidities were recorded, and the two-week pre-fracture function of the injured hip was assessed by HHS inquiry. Standard hip radiographs and computed tomography (CT) with image reconstruction were obtained for all patients with clinical suspicion for FNF. Fractures were subgrouped according to the simplified Garden classification as nondisplaced (Garden stage I or II) or displaced (Garden stage III or IV) [35] and using the vertical of the neck axis (VN) angle classification as inclined (≥ 15°) or less inclined (< 15°) [36]. Disruption of the infero-posterior wall of the femoral neck or infero-posterior comminution was confirmed on CT [37,38]. Fracture verticality was measured with a goniometer according to the modified Pauwels method [32]. In situations with high clinical suspicion, if lower extremity rotation precluded accurate measurement of fracture verticality on the preoperative radiographs, intraoperative fluoroscopic images and immediate postoperative radiographs were utilized for confirmation. The radiologic images of all patients were screened and evaluated independently by two physicians, one musculoskeletal radiologist and one orthopedic surgeon. They agreed by consensus if there were disagreements.

All operative procedures were performed by orthopedic traumatologists (H.S. and W.Z.), with more than 10 years of experience within forty-eight hours after presentation. All patients received a single dose of antibiotics at the induction of general anesthesia. Patients were positioned in supine on a traction table and fractures were reduced under fluoroscopic control. After adequate reduction, the traction table was subsequently fixed into an appropriate position [39]. As per our institution’s protocol in order to maintain optimum reduction quality, if closed traction reduction was inadequate under fluoroscopic scrutiny in two planes, the fracture must be reduced under direct exposure. Patients requiring open reduction were excluded from the study. The Haidukkewych’ criteria were utilized to assess the quality of femoral neck reduction based on immediate postoperative x-ray radiographs and CT images [12]. An excellent reduction is considered less than 2 mm of displacement (cortical displacement) and 5° of angulation (femoral neck-shaft angle and posterior tilt angle) in any plane; good reduction is 2–5 mm of displacement and /or 5–10° of angulation; fair reduction is 5–10 mm of displacement and/or 10–20° of angulation. Displacement exceeding 10 mm or angulation of 20° is considered poor [40].

**Fig. 1:**
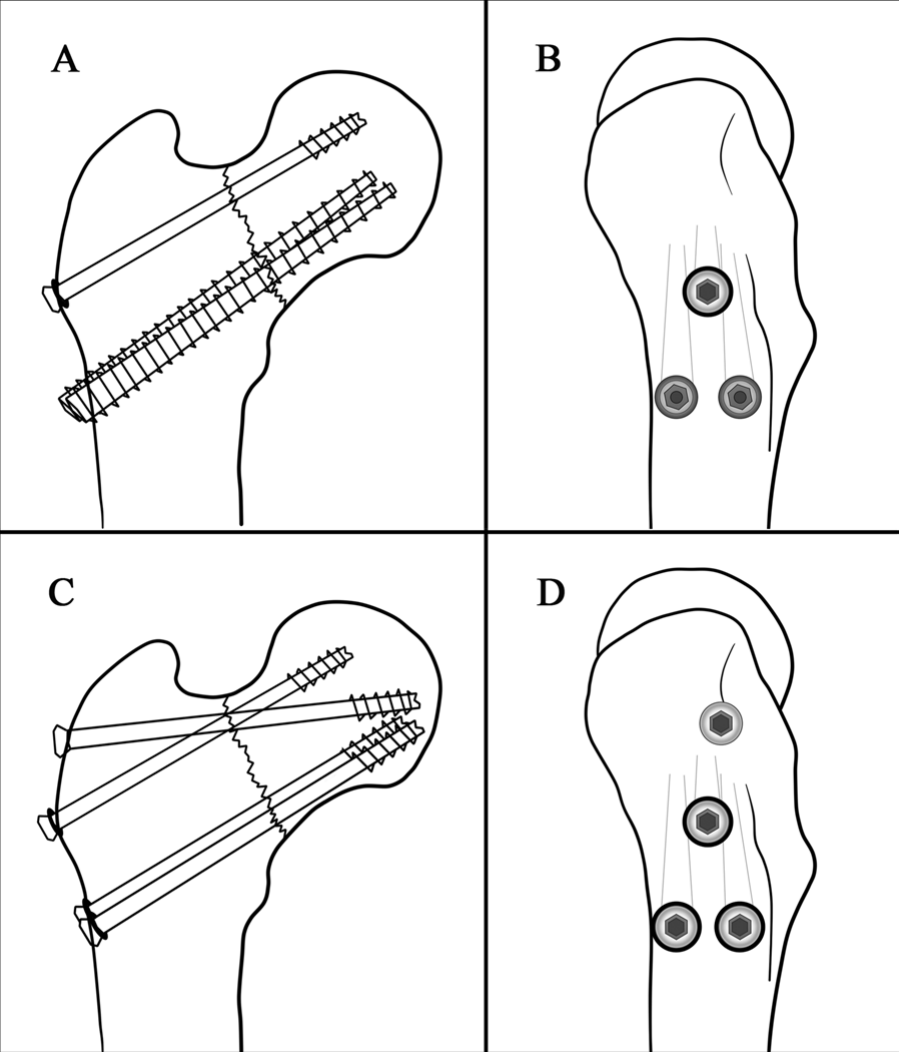
Schematic diagrams of screw configuration in two fixation strategies. **A** and **B** the CBS fixation. **C** and **D** the OPTCS fixation. **A** and **C** The left-hand side of the figure demonstrates an anteroposterior view of a vertical femoral neck fracture. **B** and **D** The right-hand side of the figure demonstrates a lateral view of the proximal femur showing regular triangle configuration

After close reduction, a sealed envelope method was used in operation room, consenting patients were randomly assigned to undergo fixation using either CBS (three parallel screws in triangle configuration, two distal FTHCSs with one proximal PTCS) or OPTCS (three parallel PTCS plus with an off-axis trochanteric transverse screw) (Fig. 1). Randomization occurred using a block procedure with a block size of four and an allocation ratio of 1:1 [41]. Due to the nature of the implants used, blinding of could only be maintained until surgery. The outcome assessor was blinded to intervention.

### Operation care

In OPTCS fixation, four guide wires were inserted. One was placed centrally, inserted from the lateral aspect of the greater trochanter and close to the proximal cortex of the femoral neck, toward the central and lower part of the femoral head at an angle perpendicular to the vertical fracture line. The other three guide wires were inserted in parallel along the femoral neck axis in a triangular configuration. The proximal guide wire of the triangle was placed posterior to the off-axis wire in order to increase posterior stability. Following this, the transverse off-axis PTCS with washer was implanted to compress the fracture site and eliminate the fracture gap. This was followed by regular triangular implantation with the same screw type (Biomet 6.5/8.0 mm, Zimmer Biomet, Warsaw, IN, USA) [42].

In CBS cohort, one PTCS screw with washer was placed in proximal-central position as the first screw in order to compress the fracture line statically. Following this, two FTHCS screws (Acutrak 6/7, Acumed, Hillsboro, OR, USA) were implanted parallelly the proximal PTCS in the distal position proximal to the level of lesser trochanter. The anteroposterior distance of the parallel screws was increased as much as possible on the lateral view to obtain greater stability [43]. In both groups, screw length was determined by intraoperative measurement.

### Postoperative rehabilitation protocol

The postoperative rehabilitation protocol was identical for both groups. Formal active physical therapy was instituted on the first day postoperatively with a focus on muscle strengthening and range of motion (ROM) exercise for the hip and knee. Patients were kept non-weight bearing for at least 8 weeks. When radiographic and clinical healing appeared to be progressing toward union, weight bearing was advanced slowly from toe touch to partial weight bearing as tolerated over the subsequent 6 weeks, at the discretion of the treating surgeon. Patients were encouraged to perform strengthening exercises until 2 years postoperatively.

### Outcome assessment

The primary outcome measure was the occurrence of complications, including fixation failure, nonunion, ANFH at 24-month follow-up. Fixation failure was defined as fracture union with > 10 mm vertical femoral neck shortening or > 10° varus collapse, without taking nonunion and ANFH into consideration [36]. Fracture nonunion was defined as gross visibility of the fracture line at 9 months postoperatively [17]. ANFH was evaluated radiographically according to Ficat criteria [44]. Postoperative CT scan or magnetic resonance imaging (MRI) for the diagnosis of nonunion or ANFH was obtained based on the clinical judgment of the treating physician. Known diameters of screws were used to correct for differences in radiograph magnification. All radiographs were independently assessed by two orthopedic surgeons (C-J. L. and S. S.) who were blinded to the objective of this study and disagreements were resolved by consensus.

Secondary outcome measures included fixation loosening, femoral neck shortening and varus collapse, HHS and HHS grade [34], and EuroQol-5 dimensions-5 levels (EQ-5D-5L) questionnaire (simplified Chinese version) at 24-month follow-up. The fixation loosening was recognized if any screw withdrawal or penetration appeared. The evaluation of femoral neck shortening was assessed according the method introduced by Zlowodzki et al. [45,46] in the vertical plane (femoral length reduction). The varus collapse was determined by the comparing the shaft-neck angles between the injury and uninjured side. Additionally, the degree of shortening and varus collapse was stratified into three categories [45]: none/mild (within 5 mm/5°), moderate (5 mm to 10 mm/5° to 10°) and severe (> 10 mm/ > 10°). Severe femoral neck shortening was defined as fixation failure, as above. The EQ-5D-5L questionnaire consists of two parts: the EQ-5D-5L descriptive system and EQ Visual analogue scale (EQ-VAS). The EQ-5D-5L descriptive system is a five-item questionnaire on patient’s general health. It covers five dimensions (mobility, self-care, usual activities, pain/discomfort, and anxiety/depression) that are divided into five levels ranging from no problems to extreme problems, which were then converted into a single index value [47]. The EQ-VAS rates a patient’s current general health status on a scale from 0 to 100 with a higher score representing a better quality of life. The national Chinese value for EQ-5D-5L has been published [48] and facilitated the calculation of quality-adjusted life years (QALYs).

Patient follow-up occurred at 1, 3, 6, 9, 12 and 24 months. Physical examination was performed, and standard radiographs were obtained at each follow-up visit. Measurements and data collection were performed during a single visit to the outpatient clinic. Interviewers (P-B. L. and Z-Y.F.) were blinded to the results of the radiological analysis. Patients unable to return for postoperative visits were assessed by these interviewers who recorded questionnaire-based outcome measures by telephone.

### Statistical analysis

An estimation of sample size was performed to detect a clinically important difference in fixation failure rates (14.3%) based on previous study [29]. Using a two-tailed distribution calculation, this analysis determined that a total sample size of 58 patients (29 per arm) would give 80% power to detect a significant difference (α = 0.05). Based on clinical experience at our institution, to allow for 10% open reduction rate and 10% loss to follow-up, the plan was to enroll 50 subjects in each group (total sample size, 100). Sample size calculations were conducted with G*Power version 3.1.8 software (Heinrich-Heine-Universität, Düsseldorf, Germany).

SPSS software (version 22.0; SPSS Inc., Chicago, Illinois) was utilized for statistical analysis. Descriptive statistics were used to characterize all variables of interest. The mean and standard deviation (SD) or the median and interquartile range (IQR) were used depending on the distribution of continuous variables, while counts and proportions were used to describe nominal data. Differences between treatment groups were compared with a Student *t* test for continuous data with a normal distribution and a Wilcoxon Mann–Whitney *U* test for continuous data with unequal variance. Pearson chi-square test or Fisher's exact chi-square test was used for comparison between groups of categorical variables.

Univariate regression analysis was performed to explore associations between fixation loosening and potential predictive variables. Using significant predictors identified in the univariate analysis, a multivariable regression model was constructed to determine the independent effect of patient or injury variables on the risk of developing fixation loosening. The prediction of fixation loosening was expressed by nomogram based on logistic regression analysis [49]. The prognostic performance of the prediction model was evaluated using a calibration curve analysis and the area under the receiver operating characteristic (ROC) curve (AUC) [50], which reflects the model’s ability to discriminate between those who will sustain a fixation loosening from those who will not. In all the statistical analyses, significance was set at 0.05.

## Results

**Fig. 2:**
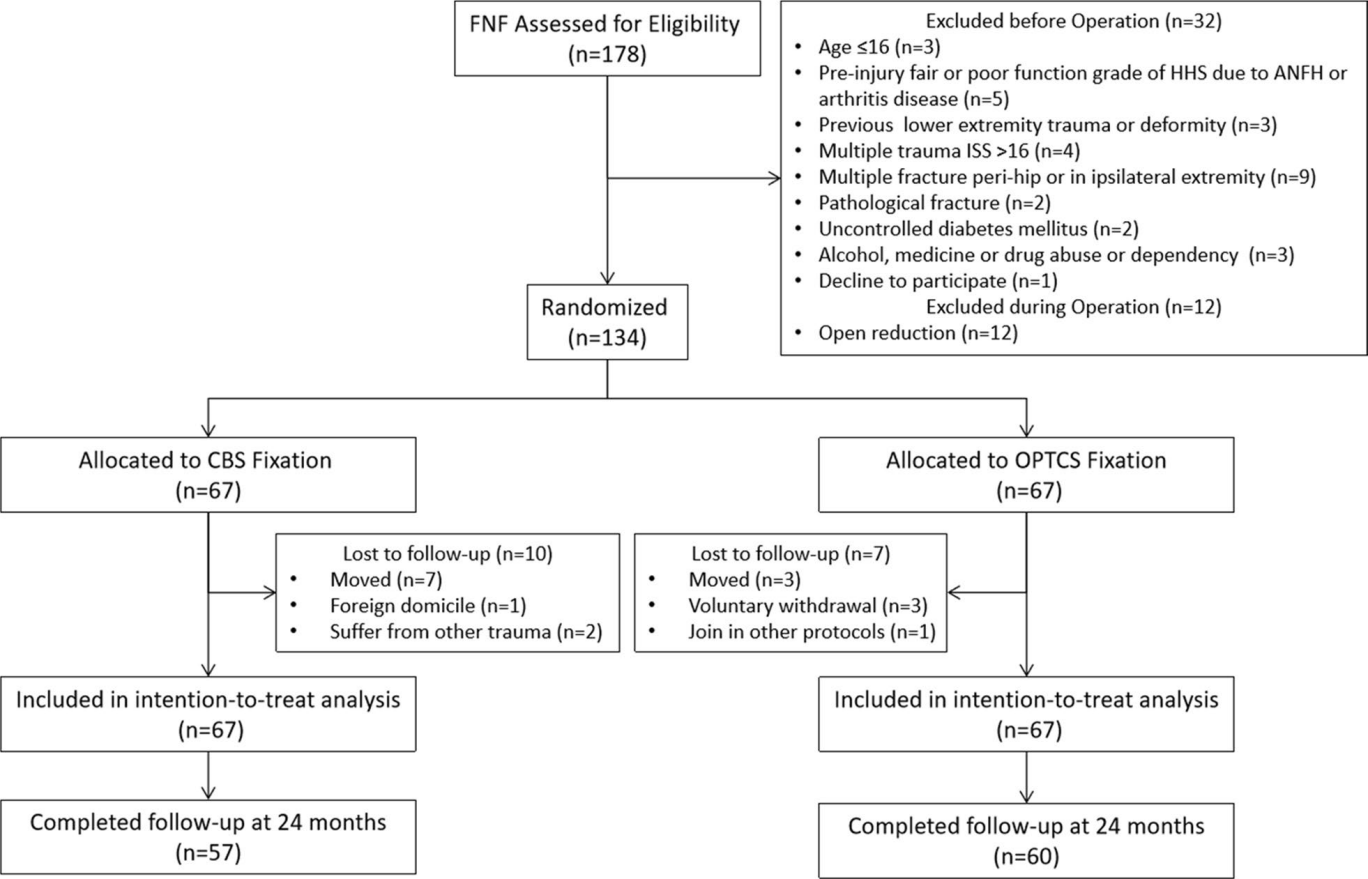
Flow diagram showing trial recruitment, randomization, and follow-up. FNF, Femoral neck fracture; HHS, Harris hip score; ANFH, avascular necrosis of femoral head; ISS, injury severity score; CBS, compressive buttress screw; OPTCS, off-axis partial threaded cannulated screw

From January 2016 through July 2017, a total of 178 patients who suffered from FNF scheduled for fixation were screened for eligibility. Before operation, 32 patients were excluded because they had not met the inclusion criteria or had met exclusion criteria. Intraoperatively, 12 patients were excluded due to irreducibility and inevitable open reduction. A total of 134 patients were randomly assigned to received CBS fixation or OPTCS fixation. After operation, 10 patients in CBS fixation were lost to follow-up, and 7 patients in OPTCS lost. A total of 57 patients in the CBS fixation group and 60 in the OPTCS fixation group were included in the pre-protocol population. The follow-up time was 31.3 ± 3.9 months in the CBS group and 31.4 ± 3 months in the OPTCS group (*p* = 0.967). Enrollment, allocation, and follow-up are summarized in Fig. 2. The baseline characteristics of patients were comparable between the two groups (all *p* > 0.05, Table 1).

**Table 1:** Baseline characteristics of the patients with femoral neck fractures treated with CBS and OPTCS fixations

Variables	CBS group (*N* = 67)	OPTCS group (*N* = 67)	*p* value
Male gender (%)	30 (52.6)	28 (46.7)	0.519
Age in years (range)	42 (33.8–54.3)	41 (30.5–54)	0.930
Right side (%)	29 (50.9)	31 (51.7)	0.932
BMI (range, Kg/m^2^)	24.4 (21.3–26.2)	25 (22.1–27.6)	0.280
Smoker (%)	16 (28.1)	18 (30.0)	0.818
Hypertension (%)	22 (38.6)	19 (31.7)	0.432
Diabetes (%)	13 (22.8)	16 (26.7)	0.629
Causes of Injury (%)			
Traffic accident	18 (31.6)	28 (46.7)	0.358
Fall	20 (35.1)	14 (23.3)	
Sport	11 (19.3)	11 (18.3)	
Pedestrian	8 (14.0)	7 (11.7)	
Singh index (%)			
Grade 6	37 (64.9)	39 (65.0)	0.967
Grade 5	15 (26.3)	15 (25.0)	
Garden classification (%)			
Nondisplaced (I–II)	16 (28.1)	23 (38.3)	0.239
Displaced (III–IV)	41 (71.9)	37 (61.7)	
VN classification (%)			
Less-inclined (< 15°)	8 (14.0)	9 (15.0)	0.882
Inclined (≥ 15°)	49 (86.0)	51 (85.0)	
Infero-posterior comminution (%)	36 (63.2)	40 (66.7)	0.691

CBS, Compression buttress screw; OPTCS, off-axial partial threaded cannulated screw; BMI, body mass index; VN, vertical of the neck axis

Patients who underwent CBS treatment had less surgery time [48 (40–55)] vs. 50 (45–60), *p* = 0.017] and less blood loss [50 (45–60) vs. 70 (57.5–90), *p* < 0.001]. There was no significant difference in bone healing time between the two groups [15 (13–16) vs. 14 (13–16), *p* = 0.340] (Table 2).

**Table 2:** Comparison of perioperative characteristics between two fixation groups

Variables	CBS group (*N* = 67)	OPTCS group (*N* = 67)	*p* value
Time to operation in days	2 (1–2)	2 (1–2)	0.265
Operating time in minutes	48 (40–55)	50 (45–60)	0.017
Blood loss in mL	50 (45–60)	70 (57.5–90)	< 0.001
Reduction Quality (%)			
Excellent	43 (75.4)	38 (63.3)	0.075
Good	12 (21.1)	22 (36.7)	
Time to union in weeks*	15 (13–16)	14 (13–16)	0.340

CBS, Compression buttress screw; OPTCS, off-axial partial threaded cannulated screw; *, the cases suffered from the nonunion were not included in statistical analysis

**Fig. 3:**
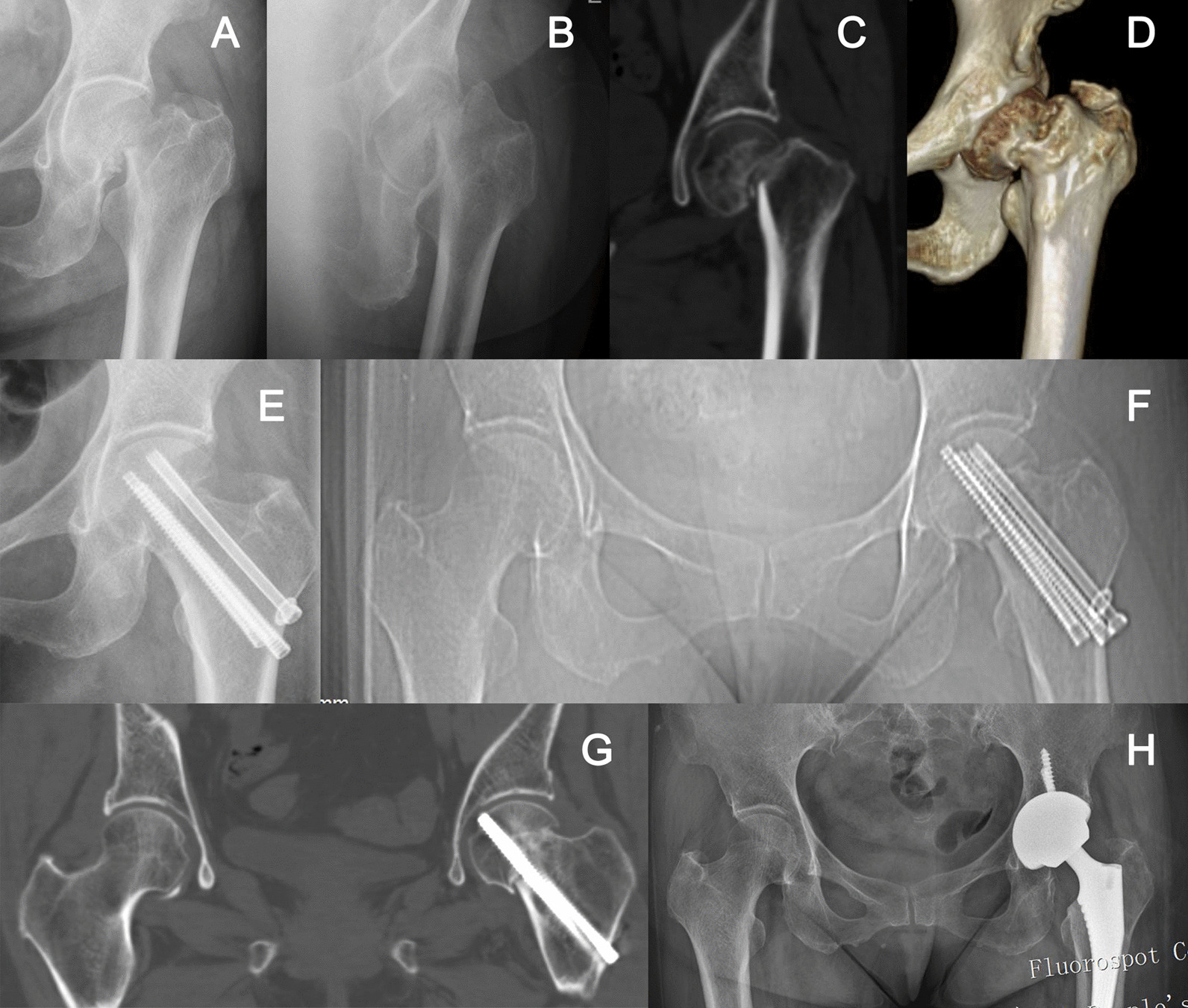
Radiographs of a 53-year-old female who sustained a FNF. **A**–**D** Anteroposterior (AP) X-ray radiographs and CT scan images of vertical displaced fracture, type Pauwels III and Garden IV; **E** AP radiograph following closed reduction and internal fixation with CBS; **F**–**G** AP radiograph and CT coronal reconstruction image revealing the nonunion with a distinctive fracture fixation loosening, medial screw migration beneath the femoral head subchondral bone in the direction of the acetabulum with slight fracture displacement; **H** The patient underwent a total hip arthroplasty due to the nonunion after 9 months following the primary internal fixation

Patients allocated to CBS fixation were less likely to develop complications than those allocated to OPTCS fixation at 24-month follow-up, including fixation failure (10.5% vs. 25.0%, *p* = 0.041), fracture nonunion (1.8% vs. 18.3%, *p* = 0.003). However, there was no statistical difference in the incidence of the ANFH (7.0% vs. 11.7%, *p* = 0.389) between the two groups (Table 3). In CBS fixation group, one patient who experienced fixation failure and nonunion simultaneously, underwent total hip arthroplasty (THA) at 12 months (Fig. 3). Another 1 patient who encountered the ANFH received the revision surgery with free vascularized fibular grafting (FVFG) and new internal fixation. In the OPTCS, 10 patients underwent various revision surgeries due to nonunion or/and ANFH.

**Table 3:** Primary and secondary outcomes in the per-protocol population

Outcomes	CBS group (*N* = 57)	OPTCS group (*N* = 60)	*p* value
Primary			
Fixation failure (%)	6 (10.5)	15 (25.0)	0.041
Fracture nonunion (%)	1 (1.8)	11 (18.3)	0.003
ANFH (%)	4 (7.0)	7 (11.7)	0.389
Secondary			
Fixation loosening (%)	11 (19.3)	35 (58.3)	< 0.001
Lateral withdrawal	11 (19.3)	35 (58.3)	< 0.001
Medial migration	1 (1.8)	0 (0.0)	0.305*
Shortening and varus collapse (%)			
None/mild	40 (70.2)	25 (41.7)	0.007
Moderate	11 (19.3)	20 (33.3)	
Severe	6 (10.5)	15 (25.0)	
HHS	93 (82.8–95)	83 (73–93)	0.001
HHS grade (%)			
Excellent	39 (68.4)	22 (36.7)	0.008
Good	3 (5.3)	7 (11.7)	
Fair	11 (19.3)	22 (36.7)	
Poor	4 (7.0)	9 (15.0)	
EQ-5D-5L	0.814 (0.609–1.000)	0.581 (0.542–0.814)	< 0.001
EQ-VAS	85 (80–90)	80 (65–90)	0.002

CBS, Compression buttress screw; OPTCS, off-axial partial threaded cannulated screw; HHS, Harris hip score; EQ-5D, EuroQol-5 dimensions-5 levels; EQ-VAS, EuroQol-visual analogue scale; ANFH, avascular necrosis of femoral head; *, Fisher’s exact test

**Fig. 4:**
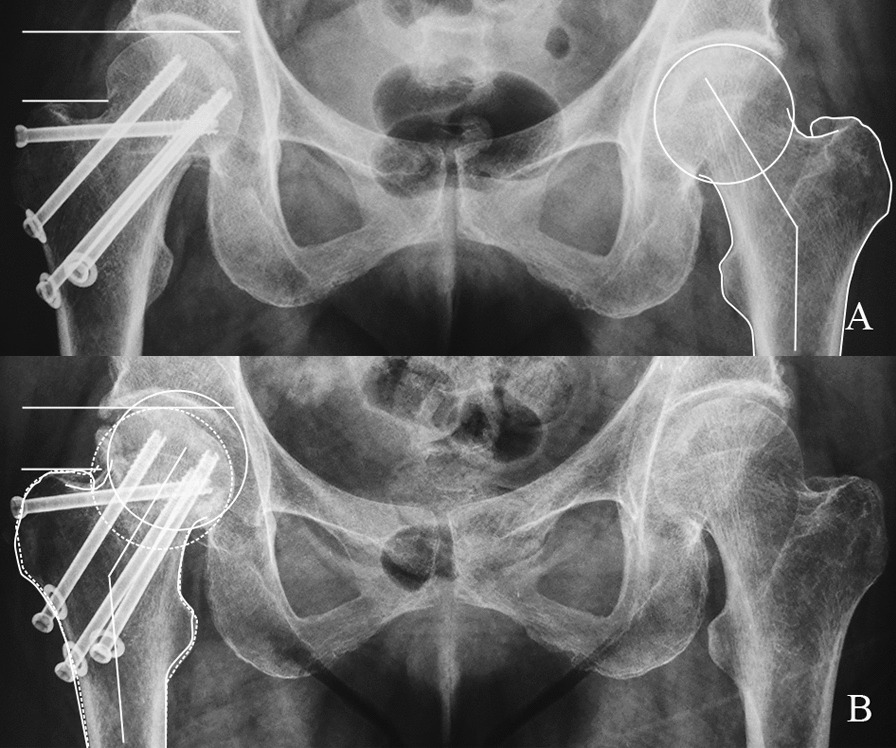
OPTCS fixation for FNF in a 47-year-old female complicated by femoral neck shortening. **A** AP pelvis radiograph demonstrating the uninjured proximal femur was outlined with the neck-shaft angle labeled. **B** 6-month postoperative radiograph demonstrates fracture union with femoral neck shortening and varus displacement. The outline of the uninjured (solid line) overlapped on the fracture side (dotted line) is provided for comparison. The screw lateral withdrawal could be detected

In both groups the most common mechanism of fixation loosening was screw lateral withdrawal; however, the OPTCS fixation group was more likely to develop screw lateral withdrawal than those receiving CBS fixation (19.3% vs. 58.3%, *p* =  < 0.001) (Fig. 4). Only one patient in the CBS fixation group suffered from the medial migration, superior cut-out of the proximal screw (Fig. 3), compared with none in the OPTCS group. The patients managed with CBS fixation showed significantly less severe femoral neck shortening and varus collapse (10.5% vs. 25.0%, *p* = 0.007). 70.2% and 19.3% patients in CBS fixation experienced none/mild and moderate shortening and carus collapse, whereas, the incidences in OPTCS group were 41.7% and 33.3% (*p* = 0.007).

With regard to the function, the CBS fixation group demonstrated significantly higher overall HHS score [93 (82.5–95) vs. 83 (73–93), *p* = 0.001] and HHS grade (*p* = 0.008) compared with the OPTCS group, although these did not meet the minimal clinically important difference between groups [51]. There were also significantly higher EQ-5D-5L [0.814 (0.609–1.000) vs. 0.581 (0.542–0.814), *p* < 0.001] and EQ-VAS [85 (80–90) vs. 80 (65–90), *p* = 0.002] scores in the CBS fixation group when compared with OPTCS (Table 3).

**Fig. 5:**
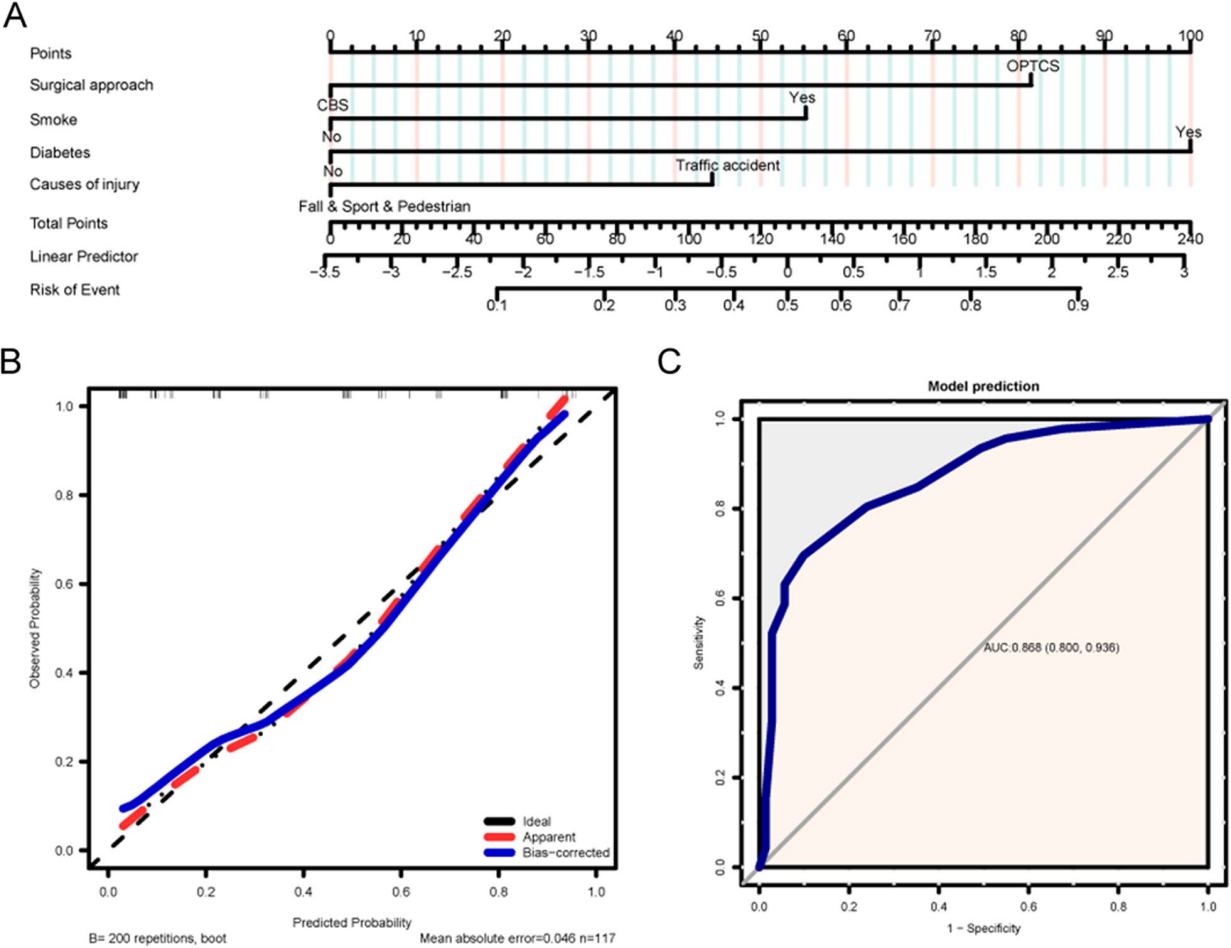
Predictive modeling based on univariate and multivariate logistic regression analysis of variables involved in fixation loosening in vertical FNF. **A** Predictive nomogram estimates of risk factors from the multivariate logistic regression analysis. **B** Using the bootstrap method, a calibration plot was constructed to compare the predicted outcome of fixation loosening to the actual outcome. **C** Area under the receiver operating characteristic curve for the predictive performance of risk factors for fixation loosening in vertical FNF was 0.868 (0.800–0.936), indicating strong predictive ability

The variables with *p* values < 0.05 in univariate analysis including surgical approach (CBS vs. OPTCS), age, smoking status, hypertension, diabetes, BMI, and cause of injury were included in the multivariate logistic regression analysis to determine the risk factors of fixation loosening in vertical FNFs (Table 4). Multivariate logistic regression analysis demonstrated surgical approach (*p* < 0.001), smoking status (*p* = 0.004), diabetes (*p* < 0.001), and cause of injury (*p* = 0.042) to be significant risk factors for fixation loosening. In order to predict the risk of fixation loosening in vertical FNF, a nomogram based on the multivariable logistic regression results was constructed (Fig. 5A). Using the bootstrap method, a calibration plot was constructed to compare the predicted outcome of fixation loosening with the actual outcome. A close fit between the predictive curve and the ideal curve indicates good predictive ability (Fig. 5B). For internal verification, the ROC showed that the resulting model had a fairly good discriminatory ability with an AUC of 0.868 (0.800–0.936) (Fig. 5C).

**Table 4:** Univariate and multivariate logistic regression analysis of patients on fixation loosening

Variables	Univariate logistic regression analysis		Multivariate logistic regression analysis	
	OR (95% CI)	*p* value	OR (95% CI)	*p* value
Surgical approach (OPTCS vs CBS)	5.85 (2.54–13.49)	< 0.001	11.62 (3.57–37.86)	< 0.001
Gender (Female vs Male)	0.55 (0.26–1.16)	0.114		
Age	1.04 (1.01–1.07)	0.022	1.01 (0.96–1.06)	0.761
Side (Right vs Left)	1.41 (0.67–2.98)	0.362		
Smoke (Yes vs No)	3.13 (1.37–7.15)	0.007	4.91 (1.67–14.47)	0.004
Hypertension (Yes vs No)	2.14 (0.98–4.66)	0.055	2.8 (0.79–9.97)	0.111
Diabetes (Yes vs No)	6.61 (2.59–16.88)	< 0.001	13.65 (3.55–52.48)	< 0.001
BMI	1.12 (1.01–1.25)	0.04	1.02 (0.88–1.18)	0.819
Causes of injury (Traffic accident vs Fall & Sport & Pedestrian)	2.09 (0.97–4.47)	0.059	3.05 (1.04–8.93)	0.042
Garden classification (Displaced vs Nondisplaced)	0.65 (0.3–1.43)	0.286		
VN classification (Inclined vs Less-inclined)	0.52 (0.19–1.47)	0.218		
Infero-posterior comminution (Yes vs No)	0.75 (0.34–1.61)	0.456		
Time to operation	0.97 (0.49–1.92)	0.924		
Operation time	1.02 (0.98–1.05)	0.285		
Blood loss	1.01 (0.99–1.02)	0.306		

OR: Odds ratio; CI, confidence interval; OPTCS, off-axial partial threaded cannulated screw; CBS, compression buttress screw; BMI, body mass index; VN, vertical of the neck axis

## Discussion

This prospective, randomized trial demonstrates that the occurrence of severe complications at 24 months is significantly less with the use of CBS fixation for the treatment of vertical FNFs in young patients when compared with OPTCS fixation. Additionally, functional scores and quality of life outcomes at 24 months are significantly improved in the CBS fixation group. We were able to identify surgical approach (CBS vs. OPTCS), smoking status, diabetes, and cause of injury (traffic accident) as predictive factors in risk of fixation loosening, which is an important reason for the complications of fixation failure, nonunion, etc., and the poor function. To our knowledge, this is the first randomized controlled trial to compare CBS to OPTCS fixation for the operative treatment of vertical FNFs in the young population.

Controversy exists with regard to the optimal screw choice and fixation configuration for the treatment of vertical FNF. Okcu and colleagues conducted a prospective, randomized study comparing the use of FTHCS with PTCS for treatment of 22 patients with FNFs encompassing all Pauwels classifications [52]. In each group, three or four screw configurations were used with the authors demonstrating that PTCS provided a shorter union time and less complication rate while providing equivalent functional results when compared with FTHCS. A second study on geriatric patients with nondisplaced Pauwels I-III FNF demonstrated similar complication rates between groups treated with three parallel FTHCSs and PTCSs [53]. However, recent studies demonstrates superior biomechanical stability and significantly reduced complication rate with the use of FTHCS for fixation for vertical FNFs when compared with PTCS [28–31]. Interestingly, a distinctive “medial screw migration” fixation loosening mechanism of three FTHCSs fixation of FNF was detected in a recent clinical study [31], highlighting the need for further study on screw configuration.

**Fig. 6:**
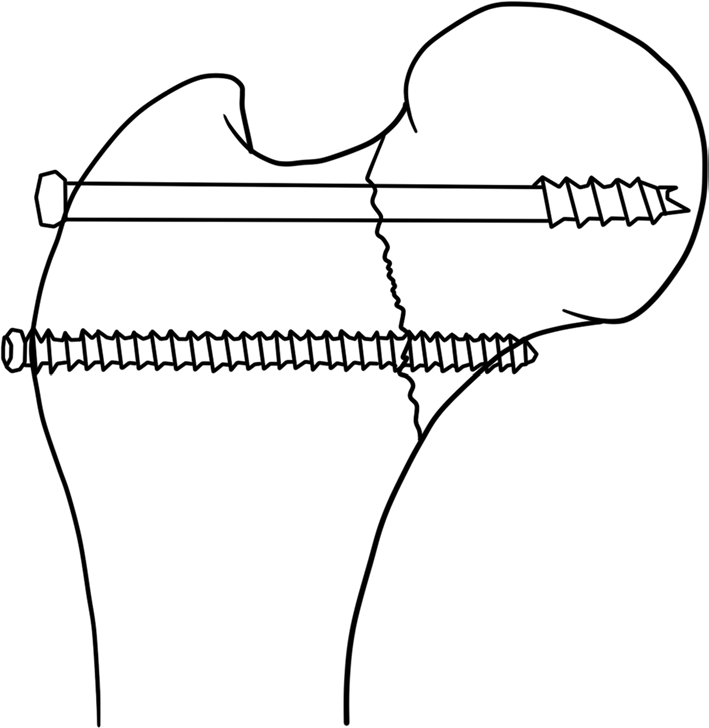
Two distinct configurations of OPTCS fixations. Trochanteric transverse screw, uni-cortical fixation of OPTCS (upper) and calcar cortex screw, bi-cortical fixation of OPTCS (lower)

Studies have demonstrated superior biomechanical stability and lower complication rates with off-axis screw configuration when compared with traditional PTCS fixation [19,24–26]. The transverse off-axis screw fixation for FNFs can be subdivided into two distinct configurations based on the level of screw implantation: the trochanteric transverse screw (TTS) and the calcar screw (CTS). The TTS is placed close to the proximal cortex, in a trajectory from lateral to inferior-medial quadrant of the femoral head (uni-cortical fixation of OPTCS, TTS-OPTCS) [17,20–22,26]. The CTS is inserted proximal to the level of lesser trochanter and fixed to the calcar cortex (bi-cortical fixation of OPTCS, CTS-OPTCS) [18,23,54,55] (Fig. 6). Although there are no clinical studies comparing these two screw configurations, a recent biomechanical study on synthetic femurs demonstrated statistically significant increases in axial stiffness and ultimate failure load for CTS when compared with TTS [56]. However, it should be noted that CTS-OPTCS fixation is not suitable for all Pauwels type III fractures, including the subcapital type and the transcervical type with medial cortex comminution. As such, TTS-OPTCS fixation was chosen for use in the present study.

A retrospective study comparing three PTCS fixation (97 patients) to those augmented with a TTS (60 patients) for vertical FNFs, demonstrated a significantly lower rate of femoral neck shortening in the TTS-OPTCS fixation cohort; however, the rates of nonunion, ANFH, and reoperation were lower in TTS-OPTCS but not statistically significant [25]. Another retrospective cohort study comparing PTCS (107 patients), TTS-OPTCS (65 patients), and dynamic hip screw (32 patients) for the treatment of vertical FNF found significantly lower fixation failure rates in the TTS-OPTCS group [19]. Taking all these into account, it is not difficult to find out that for vertical FNFs the capacity of TTS-OPTCS fixation to reduce the complication rates is limited, which is perhaps why the potency of CBS fixation for vertical FNFs was assessed and compared to the TTS-OPTCS. The results of the study proved our hypotheses.

CBS fixation, a novel fixation configuration combining FTHCS and PTCS for vertical FNF has been proposed based on clinical practice and verified by biomechanical testing [13,28–30]. Furthermore, pilot data from our group suggest that FTHCSs fixation in this new CBS configuration improves clinical outcomes in young patients with vertical FNF [29,31]. At least two inferior FTHCS in the regular triangular configuration can resist axial displacement as firmly as the non-comminuted femoral neck cortex in vertical FNF and even rebuild the lost posteromedial buttressing effect in the comminuted cases. It might be defined as “intraosseous medial buttressing effect” which is clearly distinct from the medial buttress plate fixation for treatment of vertical FNF ^13,57^.

The primary strengths of this study include the clinical relevance, strict inclusion and exclusion criteria, the large number of patients recruited, and the high rate of follow-up. Other strengths include use of a predictive nomogram based on the multivariable logistic regression analysis. As with all studies, there are limitations. First, the comparison was based on a single-center study, potentially limiting generalizability. Secondly, because the relative distribution of subjects suffered from complications, such as fixation failure and nonunion, was incomparable to that in the patients without complications in both groups, the regression analysis and nomogram for these complications could not been executed. Finally, the average follow-up time is insufficient to detect the complication of ANFH in a long-term.

In conclusion, CBS fixation for the treatment of vertical FNFs in young patients is an effective surgical technique for reducing complications, decreasing fixation loosening and femoral neck shortening/varus collapse, and improving patient’s function and quality of life when compared with OPTCS fixation. Future clinical research with larger populations should clarify if CBS fixation is superior to the traditional PTCS fixation and OPTCS, particularly bearing the biomechanical stability in mind.

## Data Availability

The datasets used and/or analyzed during the current study are available from the corresponding author on reasonable request.

## Abbreviations

CBS: Compressive buttress screw
OPTCS: Off-axial partial-threaded cannulated screw
FNF: Femoral neck fractures
ANFH: Avascular necrosis of the femoral head
HHS: Harris hip score
PTCS: Partial-threaded cancellous cannulated lag screw
FTHCS: Fully threaded headless cannulated screw
CT: Computed tomography
VN: Vertical of the neck axis
ROM: Range of motion
MRI: Magnetic resonance imaging
SD: Standard deviation
IQR: Interquartile range
ROC: Receiver operating characteristic
TTS: Trochanteric transverse screw
CTS: Calcar screw
BMI: Body mass index
